# Pills for Obstinate Neuralgia

**Published:** 1876-05

**Authors:** 


Pills for Obstinate Neuralgia.—The *Bordeaux Medical* gives the following formula for obstinate neuralgia, especially ileo-lumbar neuralgia:

℞.—Valerianate of Ammonia,
Quinine, . . . . . . aa. gr.xxx.

Make into twenty pills, and take from two to ten of them each day, increasing one pill per diem. After taking these pills for ten days, suspend their use for five days.

